# Wherever I may roam: social viscosity and kin affiliation in a wild population despite natal dispersal

**DOI:** 10.1093/beheco/arw042

**Published:** 2016-04-01

**Authors:** Ada M. Grabowska-Zhang, Camilla A. Hinde, Colin J. Garroway, Ben C. Sheldon

**Affiliations:** 1 ^a^ Edward Grey Institute, Department of Zoology, University of Oxford, South Parks Road, Oxford OX1 3PS, UK; 2 ^b^ Behavioural Ecology Group, Wageningen University, Building 122, De Elst 1, 6708 WD Wageningen, The Netherlands; 3 ^c^ Department of Biological Sciences, University of Manitoba Biological Sciences Building, 50 Sifton Road, Winnipeg, Manitoba R3T 2N2, Canada

**Keywords:** kin structure, natal dispersal, population viscosity, social behavior, vagrancy.

## Abstract

Dispersal affects the social contexts individuals experience by redistributing individuals in space, and the nature of social interactions can have important fitness consequences. During the vagrancy stage of natal dispersal, after an individual has left its natal site and before it has settled to breed, social affiliations might be predicted by opportunities to associate (e.g., distance in space and time between natal points of origin) or kin preferences. We investigated the social structure of a population of juvenile great tits (*Parus major*) and asked whether social affiliations during vagrancy were predicted by 1) the distance between natal nest-boxes, 2) synchrony in fledge dates, and 3) accounting for spatial and temporal predictors, whether siblings tended to stay together. We show that association strength was affected predominantly by spatial proximity at fledging and, to a lesser extent, temporal proximity in birth dates. Independently of spatial and temporal effects, sibling pairs associated more often than expected by chance. Our results suggest that the structure of the winter population is shaped primarily by limits to dispersal through incomplete population mixing. In addition, our results reveal kin structure, and hence the scope for fitness-related interactions between particular classes of kin. Both spatial-mediated and socially mediated population structuring can have implications for our understanding of the evolution of sociality.

## Lay Summary

When it is time to leave home and disperse from the natal area, you might expect siblings to scatter far and wide. But in the great tit, siblings associate more than unrelated birds at feeders, and not only because they start off their journey from the same nest. Complex social behaviors can evolve when relatives cluster, and knowing how those clusters persist despite dispersal is important for understanding the evolution of sociality.

## INTRODUCTION

The dispersal process consists of 3 stages: emigration, vagrancy, and settlement in the destination habitat (Ronce 2007) and encompasses aspects of physiology and behavior that can ultimately lead to gene flow in the population. Ecological factors occurring during vagrancy are likely to have important effects on settlement and the subsequent fate of individuals. However, likely due to logistical difficulties associated with monitoring dispersing individuals, the vagrancy stage of dispersal is not well understood (but see Jeanson et al. 2004; Selonen and Hanski 2011). For social species, social interactions during the vagrancy phase are likely to have particularly important consequences for aspects of subsequent settlement decisions.

Social interactions could change the proximity of relatives in space and rearrange social structure as new individuals join new social groups. For example, previous work has shown that during this period male house finches appear to choose a social environment that improves their attractiveness relative to rivals (Oh and Badyaev 2010). However, if establishing new social ties after moving to a new environment is costly (Eason and Hannon 1994), individuals may avoid changing social contexts. This could result in a socially mediated form of population viscosity during the vagrancy stage, where the movement of individuals is restricted due to social factors.

Classically defined, population viscosity (Hamilton 1964b) arises from the limited ability of an organism to move in its physical environment. This includes the restrictions that the environment poses on the final destination of the disperser, such as the availability and connectedness of suitable habitats (Wiens 2001). Social viscosity can potentially lead to similar patterns of population structure, if individuals are constrained in their movement between patches due to their social composition (Michler et al. 2011). This type of social restriction on the movement of individuals is relevant in the context of kin selection and the evolution of cooperation, as it may have a similar effect on population structure as conventionally defined viscosity. Viscosity alone may not favor kin selection for cooperative traits as it can increase competition between relatives, diminishing the inclusive fitness benefits from cooperative behaviors (Queller 1994).

Social interactions in animal populations are rarely random (Lusseau 2003; Croft et al. 2004) and have been shown to be influenced by a range of factors, including kinship (Wolf and Trillmich 2008), phenotype matching (Fowler et al. 2011), and population self-structuring (Lion and van Baalen 2008). Mechanisms that determine the social structure of populations can broadly be divided into social interaction rules and movement constraints (Sih et al. 2009). Interaction rules describe how social structure is influenced by social group composition, for example, the tendency for similar individuals to interact preferentially with each other (homophily) (McPherson et al. 2001). Movement constraints, on the other hand, describe the ways in which space use affects social structure. Spatial and temporal proximity are essential for most types of social interaction because encounters between individuals can only happen if both individuals are in the same place at the same time. Social proximity (interaction rate) can reflect spatial proximity, especially in sedentary species (McDonald 2009). However, spatial factors can also be important for highly mobile individuals, such as those undergoing dispersal. The probability of a social encounter could thus be affected by how far the individuals can travel from their point of origin as well as the decision on when to disperse.

Here, we examine factors influencing the social structure of a wild population of juvenile great tits (*Parus major*) during the vagrancy stage of natal dispersal. Outside the breeding season great tits conform to a fission–fusion social structure. Flocks of between 4 and 20 birds move through woodlands merging and splitting frequently (Hinde 1952). Our aim was to quantify the relative importance of indirect (i.e., spatial and temporal proximity of origin) and direct (kin-related) effects in determining social structure among interacting juveniles. Specifically, we investigated 1) whether associations between juvenile great tits during the vagrancy phase of dispersal could be predicted by their origin in space and time; 2) whether dispersing relatives associate at a different rate than nonrelatives, controlling for the influence of spatial and temporal proximity; and 3) whether same or different sex siblings are more likely to associate and whether the pattern of this association could be explained in terms of inbreeding avoidance.

## MATERIAL AND METHODS

### Study site and field methods

The study was conducted in Wytham Woods, Oxfordshire, UK, on a resident population of great tits that has been studied since the 1940s. The study site comprises of around 385 ha of continuous woodland, surrounded by pasture and arable land, described in more detail by Wilkin et al. (2007). The study site includes 1020 nest-boxes suitable for great tits, which have been kept at fixed locations throughout the study, save minor movements necessitated by tree falls. The boxes were digitally mapped using their GPS coordinates. All individuals used in this study originate from the Wytham population (they were ringed as nestlings in nest-boxes within Wytham Woods).

In years 2007–2009 and 2011–2013, great tits captured breeding and those born in Wytham nest-boxes were fitted with individual passive integrated transponder (PIT) tags attached to plastic leg rings. We recorded identities of breeding birds in order to obtain information on recruitment, but adult birds were not included in the dataset. The proportion of great tits in the Wytham population that were PIT tagged was estimated at circa 90% (Aplin et al. 2013). Data were collected in the winter using column feeders with 2 access points, each fitted with RFID antennae and data logging hardware.

Data collection protocols differed between 2007–2009 and 2011–2013. In the first (old) data protocol, a total of 15 feeders with loggers were rotated in a random sequence every 3–4 days between 61 fixed locations within the study site, and every 7 days in 2009. The study site was divided into 8 compartments of approximately equal size, and 7 pairs (and one single) feeders with data loggers repositioned randomly within each compartment. Every location received a logger twice every month for a period of 3–4 days in the first 2 winters and once every month for a period of 4–6 days in the third winter. In 2011, a new data protocol was implemented, where at all 61 locations, feeders automatically opened from dawn to dusk on 2 consecutive days in every seven.

Data were collected from 19 September 2007 to 25 February 2008, 11 September 2008 to 22 February 2009, and from 1 September 2009 to 17 February 2010. Under the new protocol, data were collected from 3 December 2011 to 25 February 2012, 1 December 2012 to 23 February 2013, and from 30 November 2013 to 22 February 2014.

### Social behavior and dispersal in the great tit

The great tit breeds in natural cavities but preferentially breeds in nest-boxes when these are provided. The place of birth of juveniles can therefore be easily determined. Great tits fledge from the nest at 19–25 days of age, and later disperse from their natal area as juveniles; after their first breeding season, they remain relatively sedentary (Greenwood et al. 1979; Harvey et al. 1979). Natal dispersal in the great tit is thought to play a key role in inbreeding avoidance (Szulkin and Sheldon 2008b). The breeding population structure is not random with respect to relatedness in our study population, but it is not significantly different from a null model that assumes that neighboring individuals breed together, irrespective of kinship (Szulkin et al. 2009). That means that even when dispersal is taken into account, relatives breed together more often than expected. During the process of natal dispersal, individuals are exposed to changing physical and social environments. Great tits aggregate in large, mixed species foraging flocks in the winter (Hinde 1952), and their subsequent breeding structure reflects their winter social and spatial structure (Firth et al. 2015). Dispersal in the great tit is initiated shortly after independence (Dhondt 1979) and winter ranges are expected to largely overlap with recruitment sites, because distances moved during this first dispersal wave explain a large part of the overall variation in dispersal distances (Dingemanse et al. 2003; Michler et al. 2011; van Overveld et al. 2014). Social interactions in the winter have received some recent attention in this species (e.g., Aplin et al. 2014), thanks to applying RFID technology in field studies at the time of year when birds would otherwise be more difficult to reliably identify and observe.

### Individuals and associations

Associations between individuals were deduced from temporal proximity in detections at a given logger location. Our study focused on the potential to interact, and our aim was to determine proximity between individuals, rather than direct interactions on the feeding stations. Social associations between individuals were calculated using a Gaussian mixture model that inferred group membership by detecting clusters of visits in spatiotemporal data streams (Psorakis et al. 2012). This recently developed method permits detecting associations between 2 individuals without setting arbitrary time-windows by detecting bouts of activity at feeders, which minimizes observer bias when defining association events (Psorakis et al. 2015). Associations were then quantified using the simple ratio index (SRI) (Ginsberg and Young 1992), which is an estimate of the proportion of times that birds were detected together, controlling for the proportion of times they were detected apart. We removed ~9% of individuals from our analysis due to low detection rates (less than 10 in each month), as these produce spurious extreme values of SRI.

Distances between natal nest-boxes were calculated from box coordinates. Sibling status was assigned to individuals originating from the same nest-box. A proportion of birds (ca. 13%) would therefore be assigned sibling status when they are actually half- siblings, due to the occurrence of extrapair paternity in this population (Patrick et al. 2012). However, they are still of relevance to the study because they originate from the same nest at fledging. It is possible, but less likely, that birds may associate with between-year full- or half-siblings (born to the same parents in different years), but because this study focuses on juvenile birds, these associations were not considered. We separated detections into 1-month sampling periods in order to allow for analysis of any variation in the strength of associations during the course of the winter.

As well as a measure of spatial similarity in natal origin (defined as the distance between natal nest-boxes for a pair of individuals), we also defined a measure of temporal similarity in origin, expressed as the number of days between hatching of individuals from 2 different nests. Hatching date was recorded with 1-day accuracy by frequent nest checks around the expected date of hatching (calculated from clutch size + first egg date + 12 days), and will reflect fledging date, which was not recorded directly in our study. In addition, we assigned sibling status (a dichotomous variable: yes/no) to each pair of individuals. To perform the models, we unfolded the matrices into a table where each row was a unique pair of individuals in a given month. We calculated multiple (monthly) independent networks to statistically account for variations in association strength over time.

We investigated the role of kin association by analyzing the associations of siblings of known sex in more detail. If kin are associating more than non-kin, we asked whether this was driven primarily by increased association between same-sex siblings or by increased association between opposite-sex siblings, which are at risk of inbreeding. Juveniles cannot be reliably sexed on plumage until their first molt circa 2–3 months after fledging (Svensson 1992), and because juveniles in the study were tagged as nestlings, we knew the sex of only those individuals that were subsequently captured breeding in the study site or during mist-netting sessions in the winter. Hence, for this part of the analysis, we restricted the dataset to the 48 females and 63 males that, over the course of the 6 years, recruited into the breeding population and that also had a recruited sibling. The resulting sample comprised 19 sister–sister dyads, 70 brother–sister dyads, and 59 brother–brother dyads from 75 families (67 families with 2 recruits, 8 families with 3 recruits). Birds from 3 recruit families appeared in the dataset with each of their surviving sibling (i.e., twice).

### Statistical approach

We used an information theoretic approach (Burnham and Anderson 2002) to disentangle the importance of kinship, natal distance, and asynchrony in predicting the strength of associations. There was inherent collinearity in some of our data (all siblings, and only siblings, have a natal distance of 0), and sibling status and asynchrony were also related (we assume that siblings have the same hatch dates although in this case it is possible for nonsiblings to have the same hatch dates). Collinearity causes inflation of variance in the estimated slopes in linear models. Using Akaike information criterion (AIC) model averaging reduces variance inflation and is one of ways to deal with collinearity (Freckleton 2011), provided that collinearity is not very strong and that some predictors have weak effects. All our estimates of collinearity (variance inflation factors) were close to 1, showing that collinearity was not very strong (Zuur et al. 2010), and some effect sizes were small, suggesting model averaging would be the appropriate method. We built generalized linear mixed model with binomial error structure and logit link function as our global model. The global model included our variables of interest: natal distance, sibling status, and fledging asynchrony, controlled for the effects of protocol, and for nonlinear variation in association strength over time by including quadratic effect of month. The global model also included year and individual ID as random factors.

Including pair ID as a random factor did not explain any additional variance beyond that explained by individual ID, so we did not include it in the model. We *z* transformed the predictor variables in order to standardize parameter estimates and make them directly comparable within the model (Schielzeth 2010).

All analyses were performed in R statistical environment (version 2.14.2) (R Development Core Team 2012). We used *lmer* function in lem4 package (Bates et al. 2012) to construct our model. We used the *dredge* function in MuMIn package (Barton 2013) to rank models based on AICc and *model.avg* function to average models with ΔAICc < 4 to inform our conclusions (Burnham and Anderson 2002).

We compared the associations of different sexes of sibling dyads using Kruskal–Wallis and Mann–Witney tests.

## RESULTS

### Detections and structure of associations

Across 6 years, we ringed and tagged 10196 nestlings, of which between 3% and 7% recruited to the breeding population the following year, and between 13% and 27% were recorded in the intervening winter. Figures for all years can be found in Supplementary Table S1.

The median number of detections per individual was 366 each month (range: 10–2575, interquartile range [IQR] = 210–560). The dyadic association matrix was sparse, as we observed 26144 associations, only 5% of the total 557830 possible associations of all tagged birds, and the median strength for the observed associations was 0.081 (IQR = 0.037–0.146). We observed 544 cases of associations between nest mate siblings, out of 3912 possible sibling associations (hence 14% of possible associations).

The 2 data collection protocols (2007–2010 and 2011–2014) produced the same results when analyzed separately, despite the new protocol having slightly lower mean association rates (*t* = 11.7, *P* < 0.001). We therefore decided to pool the data from 2 protocols and statistically account for that variation by including protocol as factor in our analysis. This gave our analysis greater power, adding over 400000 more individual associations at the cost of 1 df.

### Effects of kinship and origin in space and time

**Figure 1:**
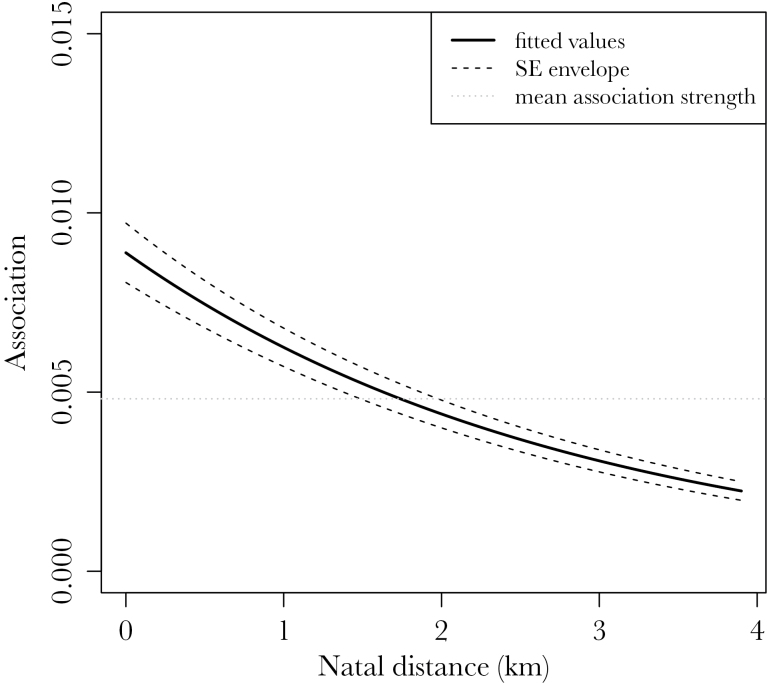
Predictions from the top model (ΔAIC = 0) show a negative effect of natal nest distance on the strength of associations. *x* axis includes full range of natal distances in the data, and *y* axis includes minimum to 3rd quartile of observed association strengths. Dashed lines are the SE envelope for the slope, and the gray dotted line is the mean association strength.

There were 6 models within ΔAICc < 4 (Table 1), and we used these for the model averaging procedure. Sibling status and natal distance were the predictors with highest relative importance of 1 in our averaged model (Table 2). Siblings associated much more than unrelated juveniles (β = 1.08, standard error [SE] = 0.12), with natal distance having a moderate, negative effect on the association rate (β = −0.486, SE = 0.043) (Figure 1). There was an expected, large, higher-order effect of month on the strength of associations, with association rates starting off low in autumn, picking up in the middle of the winter, and waning again at the end of the winter. This effect corroborates what we know of seasonal variations in foraging patterns in this species (Morse 1978). Asynchrony in fledging dates had a very small (β = 0.081, SE = 0.046) effect with low relative importance (Table 2). Protocol employed had a small negative effect (β = −0.216, SE = 0.10) that can be largely attributed to higher site coverage in the new protocol that can cause small increase in the denominator of SRI (association index). This effect had the lowest relative importance (0.63) of all variables in the averaged model (Table 2).

**Table 1:** Models explaining the strength of associations between juvenile great tits, ranked according to ΔAICc

Model	df	logLik	AICc	ΔAICc	Weight
Prot + sib + month^2^ + asy + dis	9	−9128.50	18274.99	0	0.35
Prot + sib + month^2^ + dis	8	−9130.06	18276.11	1.12	0.20
Sib + month^2^ + asy + dis	8	−9130.12	18276.24	1.25	0.19
Sib + month^2^ + dis	7	−9131.77	18277.53	2.54	0.10
Sib + asy + dis	6	−9132.85	18277.70	2.71	0.09
Prot + sib + asy + dis	7	−9132.00	18278.00	3.01	0.08

These 6 models were used in the averaging procedure. Hierarchy of terms was preserved in all models. Akaike weights indicate evidence for the model being the best within the set. All models contained individual identity (*n* = 1406) and year as random factors. Asy, hatching asynchrony; dis, natal distance; month, month from September to February; prot, data collection protocol as a factor with 2 levels; sib, sibling status (yes/no).

**Table 2:** Summary of the averaged model predicting strength of juvenile associations in the great tit

	Standardized estimate	SE	*Z* value	*P*	Relative importance
Intercept	−5.37739	0.06357	84.595	<0.001	
Dis	−0.48636	0.04334	11.223	<0.001	1
Sib	1.08103	0.12151	8.897	<0.001	1
Month	15.83454	15.97512	0.991	0.3216	n/a
Month^2^	−38.78823	16.09696	2.41	0.0160	0.83
Asy	−0.08130	0.04600	1.767	0.0772	0.70
Prot	−0.21599	0.10012	2.157	0.031	0.63

Natal distance and sibling status have the largest relative importance, suggesting they are more informative than variations in association strength in time or effects of changes data collection protocol, or similarity in hatching date. All linear terms have been *z* transformed; sibling status and protocol have been centered. Estimated variances for random terms were 0.047 for individual identity and 0.0087 for year. n/a, not applicable.

### Sibling associations

**Figure 2:**
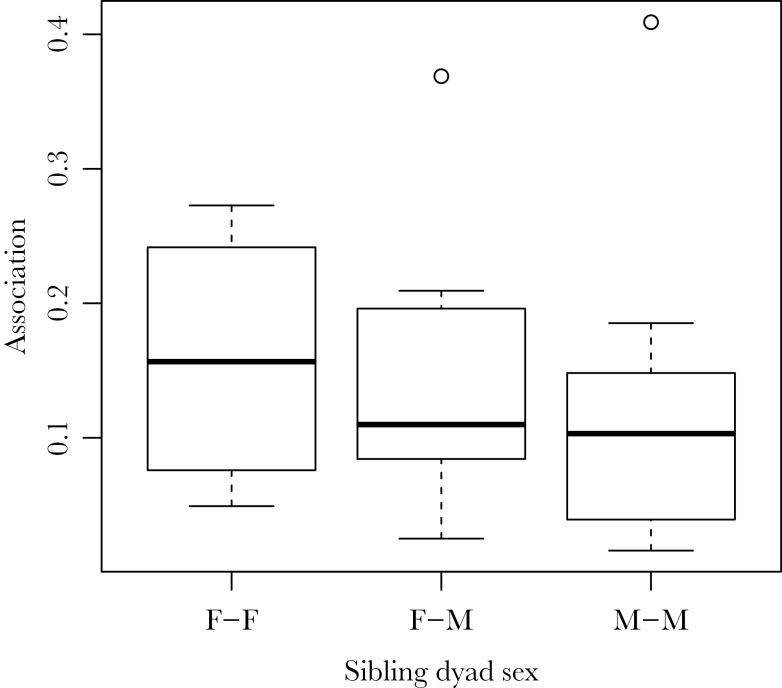
Sibling pairs of different sex compositions do not differ in their median nonzero association rates (Kruskal–Wallis test: χ^2^ = 1.59, df = 2, *P* = 0.45). “F–F” represents pairs of sisters, “F–M” are sister–brother pairs, and “M–M” are pairs of brothers. Boxes represent IQR.

We compared the association rates of siblings depending on sex. Males and females did not differ in detectability (χ^2^ = 0.0001, degrees of freedom [df] = 1, *P* = 0.9). Including all possible associations, sibling dyads of known sex did not associate at different rates (Kruskal–Wallis test: χ^2^ = 0.1793, df = 2, *P* = 0.914). The strength of nonzero associations did not differ between sibling groups (Kruskal–Wallis test: χ^2^ = 1.5938, df = 2, *P* = 0.45; Figure 2), suggesting that pairs of siblings with different sex combinations do not differ in the propensity to associate, and for pairs that do, their associations are of similar strength.

## DISCUSSION

In the winter, great tits aggregate in loose, roaming, foraging flocks that are thought to increase foraging efficiency and aid predator detection (Morse 1978). Here, we demonstrate that for dispersing juveniles, distance between natal sites limits the scope for individuals to associate socially at a feeder (Table 2). This social viscosity is likely a result of physical dispersal constraints experienced by individuals, and the distance effect confirms our expectations of limits to dispersal. Natal dispersal in the great tit is costly in terms of energy, as well as mortality risk, and in this population, the paucity of suitable habitats within a considerable distance of the study area. Most birds disperse a much shorter distance than those covered by the continuous habitat of the study site (median natal dispersal distance is 788 m for females and 528 m for males) (Szulkin and Sheldon 2008b); therefore, birds born relatively closer together will have a greater chance of temporarily residing in the same area than birds born further apart. These constraints may point to a spatial self-structuring process operating in the population (Lion and van Baalen 2008).

Notably, siblings associated more than unrelated individuals even after accounting for spatial and temporal predictors of social relationships. Siblings of known sex associated at the same rate, regardless of the sex composition of the dyad (Figure 2). We did not observe lower association rates for brother–sister pairs, suggesting no active inbreeding avoidance (Szulkin and Sheldon 2008a). A study on the Wytham population has found that brother–sister mating pairs occur more frequently than expected, and the authors suggest that strong social bonds may play a role in this maladaptive behavior (Szulkin et al. 2009). Here, we show that, like other sex combinations of siblings, brothers and sisters are indeed strongly associated in the winter, which may explain the higher than expected incidence of inbreeding. Siblings may associate because kin competition does not play a major role in the population (Lambin et al. 2001), or as a by-product of dispersing in similar directions (Matthysen et al. 2005). However, the direction of causality in this instance is a matter of speculation. Some siblings may be associating because they are dispersing in a similar direction, or they may settle closer to each other as a result of social viscosity in winter. Alternatively, both can result from behaviors occurring before full independence from parental care, such as postfledging family movements (van Overveld et al. 2011).

Asynchrony in birth dates also had a negative, albeit very small (β = −0.081, SE = 0.046), effect on the strength of associations. The small effect size indicates that this factor may be of little biological importance in our study population.

Social viscosity can have implications for the evolutionary role of population structuring. First, dispersal may not necessarily lead to dissolution of kin structure, as is frequently assumed. Here, we show sibling associations persist after accounting for spatial factors, which is reflective of kin structure in dispersing juveniles. Second, kin structure in associations can enable kin selection to operate within the population. While usually studied in social species, kin structure is widespread also in solitary and territorial animals (Sera and Gaines 1994; Stoen et al. 2005; Foerster et al. 2006) and may give rise to kin-biased behaviors under kin selection (Hatchwell 2010). Third, the evolution of cooperation, whether by the means of kin selection or not, may depend on socially mediated population structuring. Given that many models for the evolution of cooperation require a form of population viscosity (Hamilton 1964a; Taylor 1992; Grafen and Archetti 2008; Lion and van Baalen 2008), social viscosity maintained despite dispersal may be important for our understanding of evolution of cooperation and sociality in different systems.

## SUPPLEMENTARY MATERIAL

Supplementary material can be found at http://www.beheco.oxfordjournals.org/

## FUNDING

This study was supported by Natural Environment Research Council (NERC) through a DPhil scholarship to A.M.G.-Z. and by grants from NERC (NE/D011744/1) and the ERC (AdG 250164) to B.C.S.

## Supplementary Material

Supplementary Data
