# Supplementary material for: Wherever I may roam: social viscosity and kin affiliation in a wild population despite natal dispersal
Source: Behav Ecol. 2016 Apr 1;27(4):1263–8. doi: 10.1093/beheco/arw042 (PMC4943112; doi:10.1093/beheco/arw042)
Supplement: Supplementary Data [file supp_27_4_1263__index.html]

Wherever I may roam: social viscosity and kin affiliation in a wild population despite natal dispersal — Wherever I may roam: social viscosity and kin affiliation in a wild population despite natal dispersal — Supplementary Data 

# Wherever I may roam: social viscosity and kin affiliation in a wild population despite natal dispersal

## Supplementary Data

Data files

- Supplementary Data - Supplementary Data
