# Supplementary material for: Wherever I may roam: social viscosity and kin affiliation in a wild population despite natal dispersal
Source: Behav Ecol. 2016 Apr 1;27(4):1263–8. doi: 10.1093/beheco/arw042 (PMC4943112; doi:10.1093/beheco/arw042)
Supplement: Supplementary Data [file supp_arw042_Viscosity_ESM.docx]

**Online Supplementary Material**

**Table S1 Summary of numbers of birds used in the study, by year. Tagged birds are all birds that were tagged as 15 day-old nestlings in study nest boxes. Detected birds are birds recorded by loggers between September and February. Birds included in all models are restricted to those individuals that were detected more than 10 times in an individual month. Recruited birds include those birds used in analysis that were recorded breeding in the study area in the following year.**

| year | 2007 | 2008 | 2009 | 2011 | 2012 | 2013 | total |
| --- | --- | --- | --- | --- | --- | --- | --- |
| tagged | 2878 | 2097 | 1441 | 1773 | 1021 | 986 | 10196 |
| detected | 250 | 240 | 386 | 242 | 78 | 247 | 1443 |
| included | 246 | 225 | 375 | 242 | 75 | 243 | 1406 |
| recruited | 90 | 66 | 95 | 45 | 30 | 34 | 360 |
